# Angiotensin Type 1a Receptor Signaling Is Not Necessary for the Production of Reactive Oxygen Species in Polymorphonuclear Leukocytes

**DOI:** 10.5402/2012/347852

**Published:** 2012-12-04

**Authors:** Fumiko Yamato, Junji Takaya, Shoji Tsuji, Masafumi Hasui, Kazunari Kaneko

**Affiliations:** Department of Pediatrics, Kansai Medical University, Osaka, Moriguchi 570-8506, Japan

## Abstract

*Background*. Although angiotensin II (Ang II) has inflammatory effects, little is known about its role in polymorphonuclear leucocytes (PMLs). To elucidate the role of Ang II in PMLs ROS production, we examined hydrogen peroxide (H_2_O_2_), one of the ROS, and NO production in AT1a receptor knockout (AT1KO) mice. *Methods and Results*. PMLs were analyzed from Ang II type 1a receptor knockout mice (AT1KO) and C57BL/6 wild type mice. Using flow cytometry, we studied hydrogen peroxide (H_2_O_2_) production from PMLs after *Staphylococcus aureus* phagocytosis or phorbol myristate acetate (PMA) stimulation. Nitric oxide (NO) production in the AT1KO was low at basal and after phagocytosis. In the AT1KO, basal H_2_O_2_ production was low. After PMA or phagocytosis stimulation, however, H_2_O_2_ production was comparable to wild type mice. Next we studied the H_2_O_2_ production in C57BL/6 mice exposed to Ang II or saline. H_2_O_2_ production stimulated by PMA or phagocytosis did not differ between the two groups. *Conclusions*. AT1a pathway is not necessary for PMLs H_2_O_2_ production but for NO production. There was a compensatory pathway for H_2_O_2_ production other than the AT1a receptor.

## 1. Introduction

 Although angiotensin (Ang) II has been reported to have proinflammatory and oxidative effects, little is known about the correlation between Ang II and reactive oxygen species (ROS) in polymorphonuclear leukocytes (PMLs). Activation of the renin-angiotensin system and increased production of Ang II are implicated in the pathogenesis of hypertension, atherosclerosis, and cardiac hypertrophy [1]. Ang II acts through high-affinity cell surface receptors, which are linked to pathways classically associated with G-protein-coupled and tyrosine-kinase-mediated responses [2]. Ang II type 1 (AT1) receptors for Ang II have also been found in circulating neutrophils [3] and human circulating PMLs [4]; furthermore, Ang II-induced cell activation has been reported [5, 6]. One major mechanism through which Ang II induces pathological effects is regulated by the generation of superoxide (O_2_^−^) and other ROS [7]. ROS generation is mediated through the activation of NADPH oxidase in PMLs. PMLs ROS production participates in host defense by killing bacteria, but can also damage host tissues and play an important role in disease pathogenesis. 

 Nitric oxide (NO) regulates important functions of PMLs, including chemotaxis, adhesion, aggregation, apoptosis, and PMN-mediated bacterial killing or tissue damage [8]. It has been reported that rat PMLs constitutively express neural NO synthase (nNOS) mRNA and nNOS protein and exhibited spontaneous basal release of low concentrations of nitrate and nitrite anions [7]. The presence of nNOS and iNOS in rat peripheral PMLs has been well documented [7, 9]. Superoxide readily reacts with NO to produce peroxynitrite, thereby, decreasing NO levels. NO is an important mediator in a wide range of physiological and pathophysiological processes involving ROS. NO and superoxide have opposing effects on vascular tone, reacting and negating one another. ROS induced by Ang II may be involved in endothelial dysfunction through inactivation of endothelium-derived NO [10].

There are several controversial reports regarding the effect of Ang II on PMLs function [5, 11]. El Bekay et al. reported that neutrophils are highly responsive to Ang II in the context of (O_2_^−^) production [5]. To elucidate the role of Ang II in PMLs ROS production, we examined hydrogen peroxide (H_2_O_2_), one of the ROS, and NO production in AT1a receptor knockout (AT1KO) mice.

## 2. Materials and Methods

### 2.1. Mice

Eight-week-old male wild type (*n* = 6), and Ang II type 1a receptor knockout (AT1KO) mice (*n* = 4) from the same genetic background were used. C57BL/6 and AT1KO mice were obtained from Shimizu Laboratories (Kyoto, Japan). All mice were maintained on a 12-hour light/12-hour dark cycle and fed normal mouse chow. Animal care and the experimental procedure were performed in accordance with Kansai Medical University animal care facility guidelines and the National Research Council guidelines.

### 2.2. Chemicals

 2′ 7′-Dichlorofluorescein diacetate (DCFH-DA) was purchased from Eastman Kodak (Rochester, NY); phorbol myristate acetate (PMA) was purchased from Sigma Chemical (St. Louis, MO); diaminofluorescein-2 diacetate (DAF-2/DA) was purchased from Daiichi Pure Chemical (Tokyo, Japan); EDTA 4Na and magnesium-free Dulbecco's phosphate-buffered saline (PBS) were purchased from Wako Pure Chemical Industries (Osaka, Japan). PBS containing 5 mM glucose and 0.1% gelatin is denoted as PBSg. Anti-Gr-1 antibody was purchased from Miltenyi Biotec (Aubum, CA). 

### 2.3. Bacteria

*Staphylococcus aureus*, strain ATCC 25923 (kindly supplied from Shionogi Pharmaceutical Co., Osaka, Japan), was cultured for 18h in tryptic soy broth (Difco, Detroit, MI, USA) at 37°C. The bacteria were heat-killed at 60°C for 30min, washed three times with normal saline, and labeled with a 5% solution of PI (Sigma) in normal saline for 30min at room temperature in the dark. The fluorescent bacteria were washed three times with normal saline and suspended in PBS containing 5 mM glucose. The bacterial density was adjusted to the absorbance value of 2.50 at 620 nm with an UV-visible recording spectrophotometer 240 (Shimadzu, Kyoto, Japan). The number of bacteria at this density was about 2.4 × 10^9^ colony-forming units/mL. They were stored at −80°C until use [12]. PI-labeled *S. aureus* (PIS) were opsonized with pooled human AB serum (Gemini Bio-Products, Woodland, CA, USA) for 30 min at 37°C and prepared for phagocytic assay [12].

### 2.4. Measurement of PMLs NO Production

#### 2.4.1. NO Production after Phagocytosis

The measurement of NO production by PMLs following phagocytosis was described previously [13]. Briefly, 850 *μ*L of heat-killed bacterial suspension originating from *S. aureus* was added to 100 *μ*L of whole blood and 50 *μ*L of 10 *μ*M diaminofluorescein-2 diacetate (DAF-2/DA) (25 *μ*M) in PBSg in each plastic tube. The tubes were incubated with rotational agitation for 90 min at 37°C in an incubator, then 1.0 mL of 3 mM EDTA was added to terminate phagocytosis and prevent bacteria from adhering to the PMLs membranes.

### 2.5. Measurement of PMNs Hydrogen Peroxide Production

#### 2.5.1. Hydrogen Peroxide (H_**2**_O_**2**_) Production after Phagocytosis

In order to study PMLs H_2_O_2_ production, 2′ 7′-Dichlorofluorescein diacetate (DCFH-DA) was used to monitor a NADPH oxidase-mediated oxidative burst in PMLs. The measurement of H_2_O_2_ production by PMLs following phagocytosis was described previously [10]. A mixture of 100 *μ*L of heparinized whole blood, 200 *μ*L of DCFH-DA in phosphate-buffered saline PBSg (PBS containing 5 mM glucose and 0.1% gelatin), and 700 *μ*L of propidium iodide (PI)-labeled *S. aureus* suspension (1.7 × 10^9^ colony-forming units/mL) was incubated with rotational agitation for 30 min at 37°C in a shaking water bath, and then 1.0 mL of 3 mM EDTA was added to terminate phagocytosis. Erythrocytes were then removed by hypotonic lysis for 60 s. Finally, after centrifugation, each cell pellet was resuspended in 1.0 mL of 3 mM EDTA in PBSg and prepared for flow cytometry.

#### 2.5.2. H_**2**_O_**2**_ Production after Phorbol Myristate Acetate (PMA) Stimulation

A mixture of 100 *μ*L of heparinized whole blood, 200 *μ*L of DCFH-DA (10 *μ*M) in PBSg and 10 *μ*L of PMA (25 *μ*g/mL) was prepared in a plastic tube. The tube was incubated with rotational agitation for 30 min at 37°C in a shaking water bath.

### 2.6. Losartan Challenge

We have investigated the effects of treatment with AT1 receptor antagonists, losartan. C57BL/6 mice (ARB Group; *n* = 6) were administrated for 2 weeks AT1 receptor blocker (ARB), losartan (100 mg/L drinking water).

### 2.7. Infusion of Ang II

Two weeks before euthanasia, all animals were subcutaneously implanted with Alzet osmotic minipumps (model 1002; Durect Corporation, Cupertino, CA) under isoflurane anesthesia. In the Ang II and Saline Groups, 6 mice received Ang II (1000 ng/min per kg) and 6 were infused with saline, respectively.

### 2.8. Flow Cytometric Analysis

At the end of the incubation period, the sample was prepared for flow cytometric analysis. Erythrocytes were first hypotonically lyzed for 60 s. After centrifugation, each cell pellet was resuspended in 0.5 mL PBSg or EDTA-mixed PBSg. Single color fluorescence staining was analyzed using a cytofluorometer (EPICS XL II, Beckman Coulter Co., Hialeah, FL) with an Argon-ion laser (wavelength 488 nm) with System II Software. Data from 10,000 events per sample were acquired. Mean fluorescence intensity was determined after gating for granulocytes by their forward and side scatter characteristics. We confirmed that Gr-1 positive cells were more than 97% of the gating granulocytes. Gain and amplitude settings were determined according to blood samples from the same subject, allowing for establishment of reference gates for leukocyte identification. Settings were consistent throughout the study for each subject. Quantitative values for phagocytosis and hydrogen peroxide production were estimated according to mean PI and DCFH-DA fluorescence/cell, respectively.

### 2.9. Statistical Analysis

Statistical analysis was performed using JMP 6 (SAS Institute Inc., Cary, NC) software. Results were expressed as mean and standard deviation (SD). Further, the statistically significant differences among the groups were determined by subjecting the data to one way analysis of variance (ANOVA) with diet as the main effect, followed by inspection of all differences between pairs of means by Tukey's test. Differences were considered statistically significant at *P* < 0.05.

## 3. Results

### 3.1. NO Production

**Figure 1:**
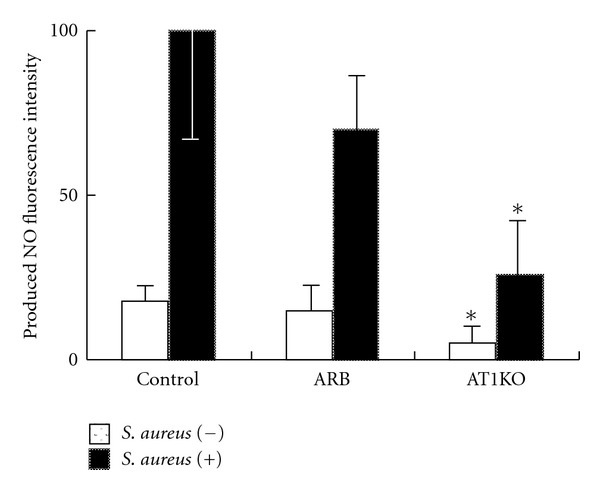
NO production in PMNs. Nitric oxide (NO) levels in polymorphonuclear leukocytes (PMLs) from C57BL/6 wild type mice (Control; *n* = 6), C57BL/6 wild type mice treated with losartan for 2 weeks (ARB; *n* = 6), and Ang II type 1a receptor knockout mice (AT1KO; *n* = 4) after the addition of *S. aureus*. *P < 0.05; compared to control mice (Control) at baseline and following the addition of *S. aureus*.

 We measured NO production from C57BL/6 wild type mice (Control Group; *n* = 6), losartan treated mice (ARB Group; *n* = 6), and AT1KO mice (AT1KO Group; *n* = 4). Animals did not differ in body weight or PMLs count. NO production in the AT1KO Group, both at baseline and following phagocytosis stimulation, was observed but lower than in the Control Group (Figure 1). NO production after phagocytosis stimulation was lower in ARB Group compared to Control Group, although the difference did not reach statistical significance. These results showed that NO production in PML is dependent on AT1a.

### 3.2. H_**2**_O_**2**_ Production

**Figure 2:**
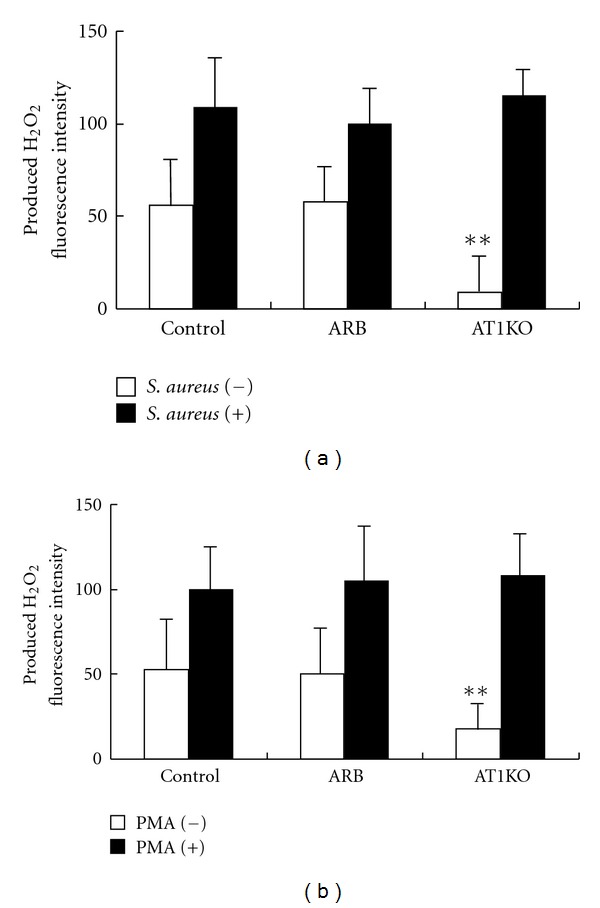
H_2_O_2_ production in PMNs. (a) Level of H_2_O_2_ in polymorphonuclear leukocytes (PMLs) from Ang II type 1a receptor knockout mice (AT1KO), C57BL/6 wild type mice (Control; *n* = 6), and C57BL/6 wild type mice treated with losartan (ARB; *n* = 6) after addition of *S. aureus*. **P < 0.01; compared to control mice (Control) at baseline. (b) H_2_O_2_ levels in polymorphonuclear leukocytes (PMLs) from Ang II type 1a receptor knockout mice (AT1KO; *n* = 4), C57BL/6 wild type mice (Control; *n* = 6), and C57BL/6 wild type mice treated with losartan (ARB; *n* = 6) after phorbol myristate acetate (PMA) stimulation. **P < 0.01; compared to wild-type mice (Control) at baseline.

Basal H_2_O_2_ production in the AT1KO Group was low (Figures 2(a) and 2(b)). After *S. aureus* phagocytosis or PMA stimulation, AT1KO mice could produce equivalent amounts of H_2_O_2_ compared to control mice (Figures 2(a) and 2(b)). These results showed that PMLs can produce H_2_O_2_ without AT1a after stimulation. There was a compensatory pathway for H_2_O_2_ production other than the AT1a receptor.

**Figure 3:**
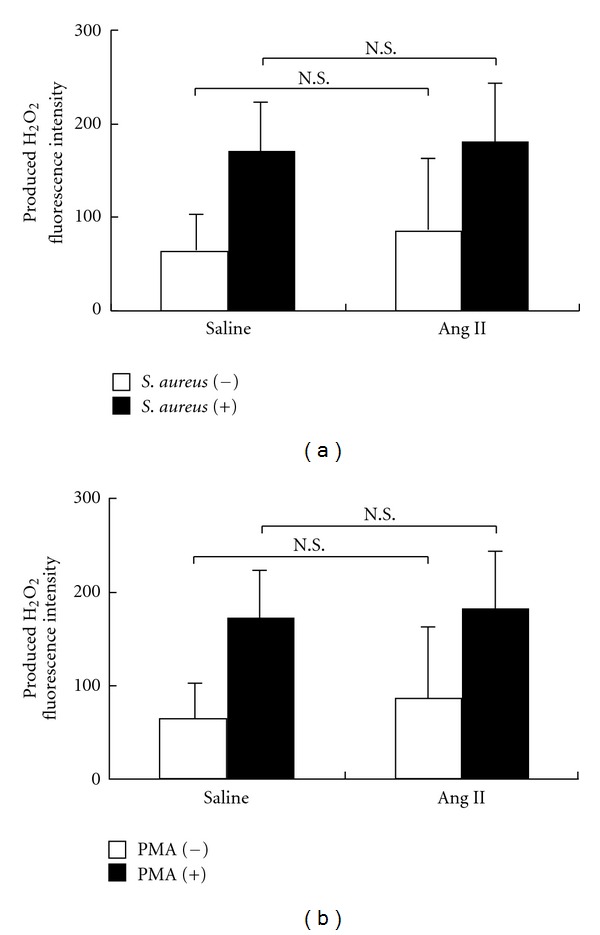
H_2_O_2_ production in PMNs. (a) H_2_O_2_ levels in polymorphonuclear leukocytes (PMLs) from mice after phorbol myristate acetate (PMA) stimulation. No significant difference was observed between the angiotensin II (Ang II) and saline exposed mice (*n* = 6 of each group). (b) Level of H_2_O_2_ in polymorphonuclear leukocytes (PMLs) from mice after addition of *S. aureus*. No significant difference was observed between the angiotensin II (Ang II) and saline exposed mice (*n* = 6 of each group).

Next, to determine if Ang II affected the production of H_2_O_2_, we tested the model mice infused Ang II for 2 weeks by osmotic pump (Ang II Group). Animals did not differ in body weight or PML count between two groups. H_2_O_2_ production stimulated by PMA or phagocytosis did not differ between the Ang II and Saline Groups (Figures 3(a) and 3(b)).

## 4. Discussion

NO production in the AT1KO Group, both at baseline and following phagocytosis stimulation, was lower than the Control Group. This result showed that AT1a pathway plays a significant role in NO production from PMLs. The presence of nNOS and iNOS in rat peripheral PMLs has been well documented [7, 9, 14]. iNOS produces large amount of NO, it could be therefore important armor to intruders during phagocytosis. Only a small percentage of AT1KO mice survive to weaning compared to wild type mice because of inflammatory problem as well as lower blood pressure or renal malfunction.

NO production after phagocytosis was lower in ARB Group compared to Control Group. In ARB Group, AT1 receptor was partially blocked *in vivo* and NO production was decreased. AT1a receptor plays a significant role in mediating NO production after phagocytosis stimulation. 

A large number of studies have demonstrated that AT II is involved in key events of the inflammatory process. Phagocyte NADPH oxidase or respiratory burst oxidase is a well-characterized reactive oxygen species-generating system that catalyzes the 1-electron reduction of oxygen to superoxide radical (O_2_^−^). Ang II highly stimulates endogeneous and extracellular (O_2_^−^) production in human neutrophils, consistent with the translocation to the cell membrane of the cytosolic components of NADPH oxidase [5]. 

Ang II acts through binding to specific cellular receptors, of which AT1 and AT2 are the best characterized [15]. AT1 receptors mediate many important cardiovascular responses, such as vasoconstriction, vascular, and cardiac remodeling. In mouse aortic rings, Ang II increases aortic protein levels of NADPH oxidase component with increased (O_2_^−^) production [16]. After exposure to Ang II, the elevation of superoxide production occurs through AT1 receptor mediated activation in NADPH oxidase of the coronary arterioles, renal cortices, and human umbilical vein endothelial cells. We tested whether PMLs from AT1KO mouse can produce H_2_O_2_.

To our surprise, AT1KO mice produced H_2_O_2_ from PMLs equivalent amount of H_2_O_2_ compared to control mice. AT1a pathway is not necessary for PMLs H_2_O_2_ production. In addition, our results show that PMLs exposed to Ang II for 2 weeks had no effects on H_2_O_2_ production after PMA or phagocytosis stimulation. It has been hypothesized that activation of the AT2-receptor may be antagonistic to AT1-receptor activation. AT2 receptor may play a counter-regulatory protective role mediated by bradykinin and NO [17]. There are several controversial reports regarding the effect of Ang II on PMLs function [5, 11]. El Bekay et al. reported that neutrophils are highly responsive to Ang II in the context of (O_2_^−^) production [5]. Ang II upregulates several subunits of NADPH oxidase. However most evidence suggests that assembly of NADPH oxidase onto cell membranes is initiated by Rac-1, which is activated by Ang II binding to AT1 receptor. This study demonstrated for the first time that AT1a receptor is not essential for H_2_O_2_ production from PMLs.

In conclusion, we have demonstrated that AT1a pathway plays a significant role in NO production from PMLs. However, AT1KO mice can produce H_2_O_2_. The AT1a pathway is not necessary for PMLs H_2_O_2_ production.
